# The Molecular Clockwork of the Fire Ant *Solenopsis invicta*

**DOI:** 10.1371/journal.pone.0045715

**Published:** 2012-11-13

**Authors:** Krista K. Ingram, Alexander Kutowoi, Yannick Wurm, DeWayne Shoemaker, Rudolf Meier, Guy Bloch

**Affiliations:** 1 Department of Biology, Colgate University, Hamilton, New York, United States of America; 2 Department of Ecology, Evolution and Behavior, The A. Silberman Institute of Life Sciences, Hebrew University of Jerusalem, Jerusalem, Israel; 3 Department of Ecology and Evolution, University of Lausanne, Lausanne, Switzerland; 4 School of Biological and Chemical Sciences, Queen Mary University of London, London, United Kingdom; 5 Agricultural Research Service, United States Department of Agriculture, Gainesville, Florida, United States of America; 6 Department of Biological Sciences, National University of Singapore, Singapore, Singapore; Field Museum of Natural History, United States of America

## Abstract

The circadian clock is a core molecular mechanism that allows organisms to anticipate daily environmental changes and adapt the timing of behaviors to maximize efficiency. In social insects, the ability to maintain the appropriate temporal order is thought to improve colony efficiency and fitness. We used the newly sequenced fire ant (*Solenopsis invicta*) genome to characterize the first ant circadian clock. Our results reveal that the fire ant clock is similar to the clock of the honeybee, a social insect with an independent evolutionary origin of sociality. Gene trees for the eight core clock genes, *period*, *cycle*, *clock*, *cryptochrome-m*, *timeout*, *vrille*, *par domain protein 1* & *clockwork orange*, show ant species grouping closely with honeybees and *Nasonia* wasps as an outgroup to the social Hymenoptera. Expression patterns for these genes suggest that the ant clock functions similar to the honeybee clock, with *period* and *cry-m* mRNA levels increasing during the night and *cycle* and *clockwork orange* mRNAs cycling approximately anti-phase to *period*. Gene models for five of these genes also parallel honeybee models. In particular, the single ant *cryptochrome* is an ortholog of the mammalian-type (cry-m), rather than *Drosophila*-like protein (cry-d). Additionally, we find a conserved VPIFAL C-tail region in *clockwork orange* shared by insects but absent in vertebrates. Overall, our characterization of the ant clock demonstrates that two social insect lineages, ants and bees, share a similar, mammalian-like circadian clock. This study represents the first characterization of clock genes in an ant and is a key step towards understanding socially-regulated plasticity in circadian rhythms by facilitating comparative studies on the organization of circadian clockwork.

## Introduction

The circadian clock is a core molecular mechanism that allows organisms to anticipate daily environmental changes and adapt the timing of behaviors to maximize their efficiency. Although the components of the clock are largely conserved across a broad range of species, there is appreciable diversity in clock structure and function, particularly in insects [1]–[4]. The basic model of the molecular clock in *Drosophila* consists of positive and negative feedback loops involving a principle suite of canonical “clock genes” inside pacemaker cells [5]. The protein products of *clock (Clk)* and *cycle (Cyc)* genes interact and form a complex that bind to E-box elements in regulatory sequences of the *period (Per)* and *timeless (Tim)* gene promoter regions to activate transcription. *Per* and *Tim* mRNA accumulate in the cytoplasm during the night and the protein products enter the nucleus and bind to the CLK/CYC complex, inhibiting further transcription. On exposure to light, *Drosophila*-type *cryptochrome* (CRY-d) promotes rapid degradation of TIM that renders PER unstable. PER is eventually degraded, releasing the inhibition of transcription [6]–[9]. The activity of CRY-d allows the period and phase of the clock to adjust to changes in photoperiod [10]. Input pathways of the circadian clock respond to both environmental stimuli (including light and temperature) as well as social stimuli [8]. In fact, recent work has shown that the context of the social environment plays a major role in circadian rhythmicity in social insects [11]–[13]. Mapping the diversity in insect clock structure on an evolutionary backdrop of organisms that vary in key life history characteristics including social structure may reveal how evolution has shaped the various functions of the clock components [1], [3], [4], [11], [12], [14].

In social insects, the ability to maintain the correct temporal order is not only important for individual success, but also plays a significant role in the success of the colony. Circadian timing is important for foraging activities, sun-compass navigation, timing of mating flights, and synchronization of individuals and the organization of colony tasks, such as nest maintenance [11], [14], [15]. Previous work has demonstrated that the circadian clock is associated with the division of labor in honeybees, bumble bees and ants, suggesting that the circadian clock is important for the social organization of insect societies [12], [15]–[19]. The evolution of task-related plasticity in social insect colonies is thought to enhance task specialization and to improve colony efficiency; brood care workers have no circadian rhythms and are active around the clock whereas foragers have strong circadian rhythms with peaks of activity accurately timed to the external environments that they encounter [14], [15], [18], [19]. Chronobiological plasticity has been documented in species that have evolved sociality independently (ants and bees) and in species that differ in organization of social structure (age-related task specialization in honey bees and size-related task specialization in bumble bees), suggesting that the evolution of clock-related plasticity in social insects is functionally significant [14], [17]. Recent evidence that social factors can also affect the ontogeny of circadian rhythms and social synchronization in honeybees further suggests that the evolution of social insect societies has influenced the circadian clock in these species [11], [12], [14]. Given this evidence for a significant interplay between the circadian clock and social organization is limited to bees, it is not clear whether these findings represent a general genuine influence of social evolution on the circadian system or a unique set of traits that are limited to the honey bee. To understand the link between circadian clocks and sociality, we need to study additional social species, including hemimetabolous and holometabous insects, that differ in life history traits.

Ants make an attractive model system for studying sociochronobiology because they display a remarkable diversity in ecology, morphology, behavior and life history characteristics and represent an independent evolution of sociality within the Hymenoptera [20]. All ants are social and most show a highly derived form of sociality with a sterile worker caste characterized by behavioral specialization in different morphological [castes] or behavioral phenotypes [tasks] [21], [22]. Colonies have no central control and interactions between workers in the form of antennal contacts and chemical detection determine decision-making and adjustments in worker behavior and task allocation [23], [25]. In addition, the maturation of workers through different tasks occurs on the order of weeks to months in many ant species [20], rather than days in honey bees. Thus, the ontogeny of circadian rhythms may be slower in these ant species. Differences in the underlying social framework of ants and honey bees provide an ideal comparative framework for understanding the evolution of social clocks and the link between molecular mechanisms of circadian rhythm and social behavior.

The molecular dissection of the honeybee social clock revealed a clock mechanism that according to at least five lines of evidence is more similar to the mammalian model rather than to that of *Drosophila* [2], [26]. First, the honeybee genome encodes a mammalian-type cryptochrome *(Cry-m)* rather than *Cry-d*, the ortholog found in *Drosophila*. *Cry-m* is not sensitive to light in-vitro and thus it is thought that it does not function in the photic input pathway to the clock, an essential function of *Cry-d* in *Drosophila* [5]. Second, the honeybee also lacks an ortholog to *timeless*, an essential gene for clock function in *Drosophila*. Third, the CYC protein contains a transactivation domain in honeybees that is found in *Drosophila* CLK [2], [14]. Fourth, in foraging bees that show strong circadian rhythms, brain mRNA levels of *Cry-m* and *Per* oscillate with a similar phase in both LD and DD conditions and levels of *Cyc* mRNA oscillate anti-phase to *Per* [2], [27]. In contrast to *Drosophila*, the honey bee CLK protein does not contain a transactivation domain and the *Clk* gene does not oscillate. Finally, expression patterns of *amCyc*, *amCry*, *amClk* and *amTim2 (*the honeybee ortholog to the *Drosophila* gene, *timeout)* in honeybee brains are more similar to mammals than *Drosophila*, suggesting that the honeybee clock, in many ways, functions similar to mammalian clocks [2], [14], [26].

Here, we describe for the first time the molecular clockwork for an ant species, the fire ant *Soleopsis invicta*, a social insect pest of great ecological and economic importance [28], [29]. Using the recent fire ant genome [30], we characterize eight putative principle clock genes, *period (SiPer), cycle (SiCyc)*, *clock (SiClk)*, *cryptochrome-m (SiCry)*, *timeout (SiTim)*, *vrille (SiVri)*, *par domain protein 1* (*SiPdp1)* and the recently discovered *clockwork orange* gene (*SiCwo*) [31]–[33]. These genes were selected because they have well-documented functions in insect and/or mammalian clocks [5], [6]. We use phylogenetic analyses to establish orthology/paralogy of the eight genes across insects and use qPCR analysis to determine how the clock mechanism is functioning at the molecular level. We develop gene models for five genes in order to determine the degree of conservation for domains present in the ant clock and shared with mammalian and *Drosophila* models. In addition, we describe a novel domain shared across insect orthologs of *clockwork orange* that is absent in vertebrates.

## Results

**Figure 1:**
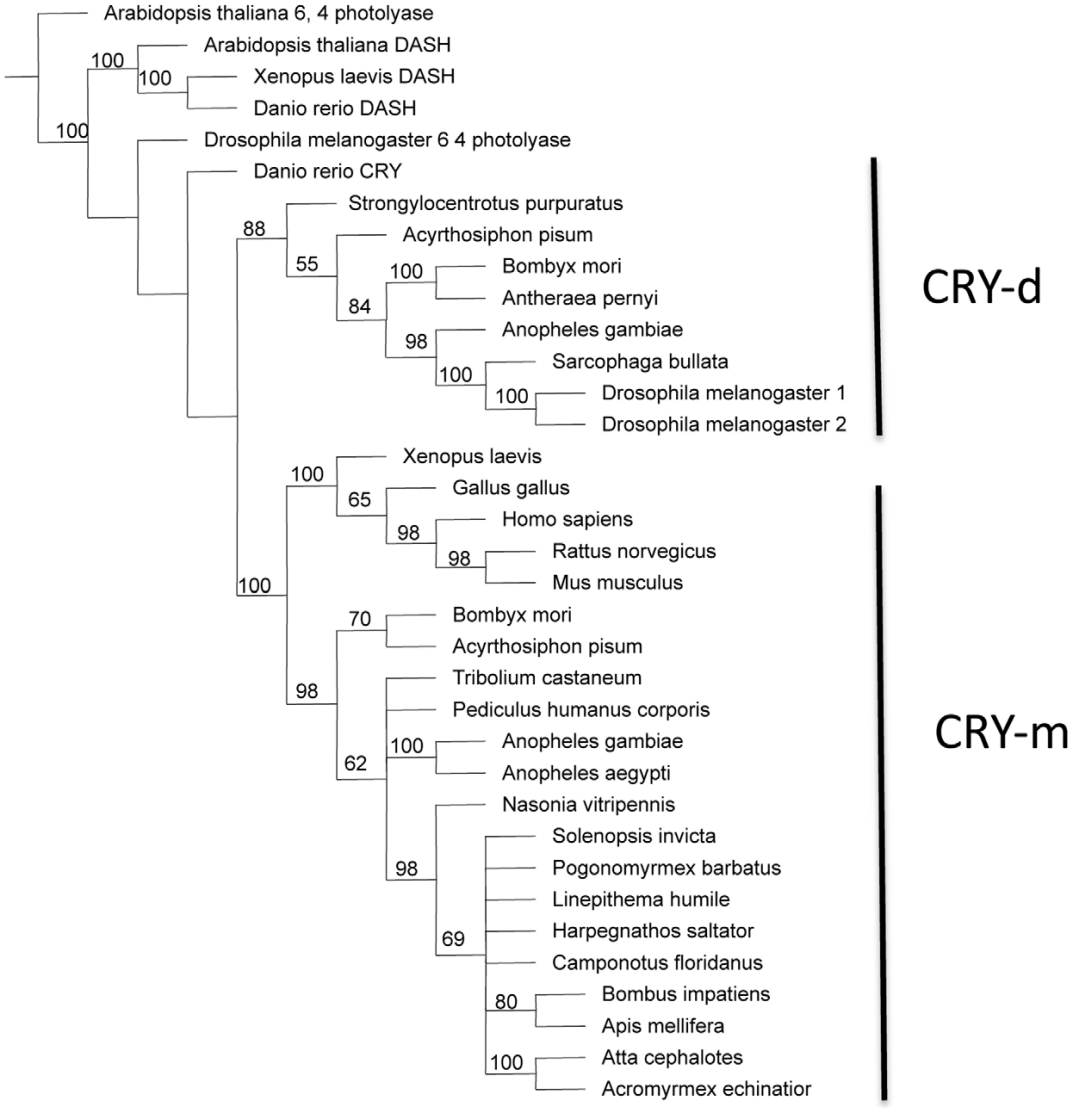
Parsimony tree for *cryptochrome* gene family. Shown is a consensus tree with bootstrap values from 250 replicates. Outgroups include orthologs of photolyase and DASH. Cryptochromes segregate into two clades, *Drosophila*-like (CRY-d, also known as "insect CRY1") proteins and mammalian-like (CRY-m, also known as "insect CRY2") proteins. CRY-d proteins are light sensitive with no transcriptive repressive activity whereas CRY-m proteins are light insensitive and may function as the main transcriptional repressors of the core clockwork [1]. All of the Hymenopterans (bees, ants, and wasps) have only the *Cry-m* genes.

**Figure 2:**
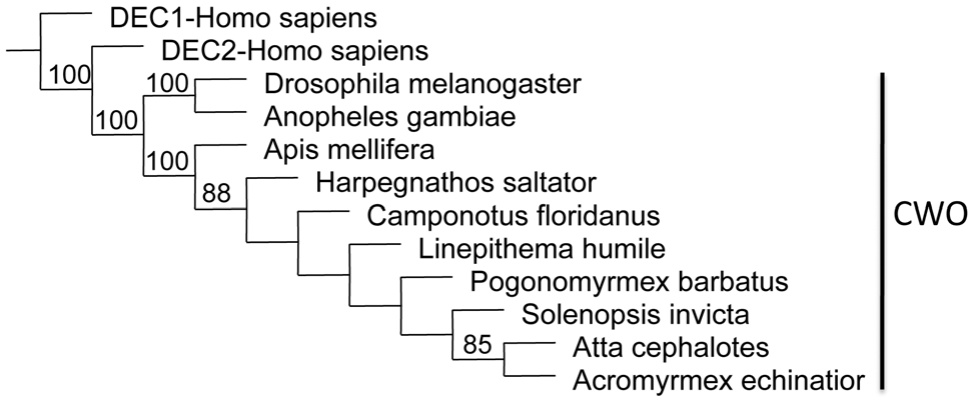
Parsimony tree for *clockwork orange* orthologs. Shown is a consensus tree with bootstrap values from 250 replicates. Insect CWO orthologs clearly separate from mammalian orthologs *Dec1* & *Dec2*.

Phylogenetic analyses of the principle clock genes show concordant patterns and confirm orthology. For each gene, orthologs from all ant species either form a monophyletic group or cluster in a polytomy with other Aculeata (bees, ants and wasps). In most analyses (with the exception of *cycle*), *Nasonia*, the non-social parasitic wasp, is the sister group to the Aculeata (Figure S1). On the tree for *cryptochrome,* the bumblebees (*Bombus impatiens*) and honeybees (*Apis mellifera*) form a clade that is nested within the Formicidae (Figure 1). The *Cry* orthologs found in the Hymenoptera clearly group with *Cry2/Cry-m* (mammalian-like *Cry*) with the *Drosophila*-like *Cry1/Cry-d* being only distantly related. The first phylogeny for *clockwork orange* in insects shows similar relationships as the other core clock genes and also shows the relationship between insect CWO and mammalian orthologs DEC1 and DEC2 (Figure 2).

**Figure 3:**
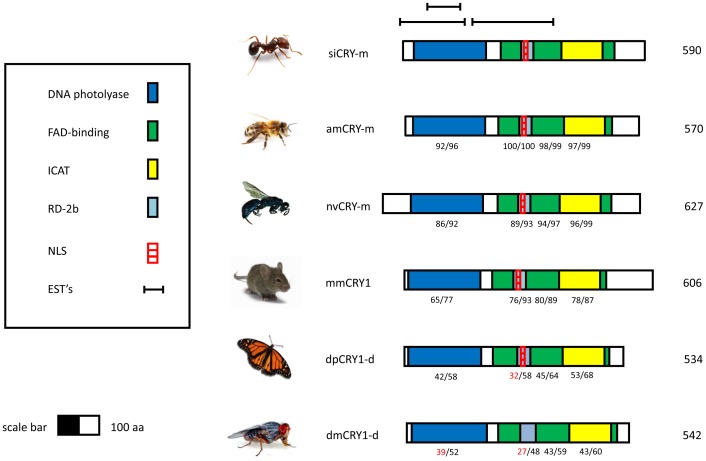
Schematic models for CRY proteins from various animals. The protein models depicted are from the fire ant *Solenopsis invicta* (siCRY), the Western honey bee *Apis mellifera* (amCRY-m), the fruit fly *Drosophila melanogaster* (dmCRY), the Monarch butterfly *Danaus plexippus* (dpCRY1) the jewel wasp *Nasonia vitripennis* (nvCRY) and the domestic mouse *Mus musculus* (mmCRY1). Highlighted areas on the diagrams represent putative functional domains and motifs. The numbers below the domains indicate percents of identity/similarity to corresponding sequences on the protein of the fire ant. The numbers at the end of each diagram indicate the predicted protein size (number of amino acid residues). The protein domains on the CRY sequence are:. • FAD binding. Proteins containing this domain are photolyases (DNA repair enzymes) or function as blue light photoreceptors (Pfam domain accession number: PF03441). • DNA photolyase. This domain is an evolutionary conserved protein domain from bacteria to mammals. It binds to UV-damaged DNA containing pyrimidine dimers and, upon absorbing a near-UV photon 300 to 500 nm, breaks the cyclobutane ring joining the two pyrimidines of the dimer (Pfam domain accession number: PF00875). • ICAT - Inhibition CLOCK-ARNTL Transcription. A domain required for the inhibition of CLOCK-ARNTL-mediated transcription (Swiss-Prot record of mmCRY1 accession number: P97784). • RD-2b – A domain defined by [58] based on studies with the clock proteins of the zebrafish. The domain is necessary for nuclear localization and the repression of CLOCK: BMAL-mediated transcription. • NLS - Nuclear localization signal in the RD-2b region, following [58]. • EST - Expressed sequence tags.

The gene models for *clock, cryptochrome, cycle* and *period* for *Solenopsis* contain all of the conserved domains and binding regions found in insect species with high amino acid similarity to *Apis* in conserved regions (Figure S2). In the *cryptochrome*-m model, the fire ant ortholog, *SiCry-m,* clearly contains the NLS (nuclear localization signal) region found in mammalian-like cryptochromes that are absent in *Drosophila*-like cryptochromes (Figure 3). In addition, the amino acid sequence surrounding this region has low similarity to *Drosophila*-like cryptochromes relative to mammalian-like cryptochromes.

**Figure 4:**
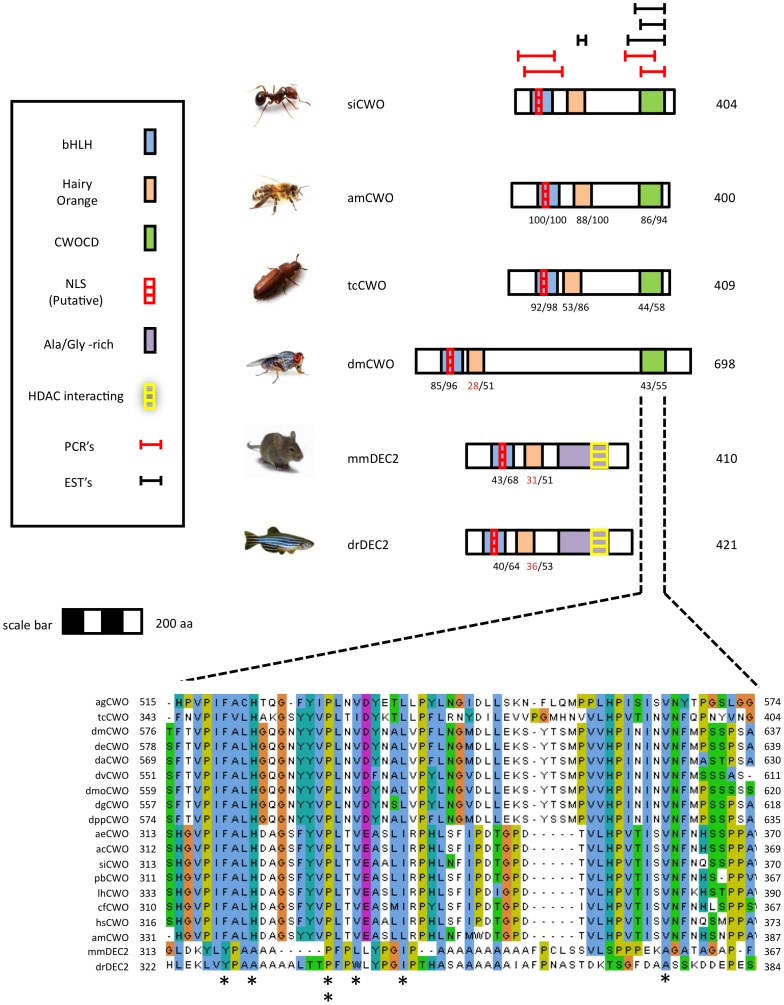
Schematic models for Clockwork Orange (CWO) proteins from various insects. The depicted models are for the fire ant *Solenopsis invicta* (siCWO), the honey bee *Apis mellifera* (amCWO), the fruit fly *Drosophila melanogaster* (dmCWO), Red Flour Beetle *Tribolium castaneum* (tcCWO). Also shown are related proteins from the house mouse *Mus musculus* (mmDEC2) and zebrafish *Danio rerio* (drDEC2). Highlighted areas on diagrams represent putative functional domains and motifs. For more details see legend to Fig. 3. Inset shows a CLUSTALW multiple sequence alignment of a new conserved domain discovered on the CWO protein sequence that we termed 'Clockwork Orange C-tail Domain' (CWOCD) the CLUSTALW alignment includes several additional CWO proteins from drosophilid and ant species (see Table S3). Asterisks in the bottom of alignment indicate amino acids conserved between insects and vertebrates. Alignments were generated with CLUSTALW and colored with JalView according to the default CLUSTALX convention. • bHLH - Basic-helix-loop-helix. Proteins containing this domain are typically dimeric transcription factors, each with one helix containing basic amino acid residues that facilitate DNA binding to an E-box. (Pfam domain accession number PF00010.). • CWOCD - Clockwork orange C-tail domain. • Hairy Orange - The Orange domain is found in the *Drosophila* proteins Hesr-1, Hairy, and Enhancer of Split. The Orange domain is proposed to mediate specific protein-protein interaction that confers specificity among members of the Hairy/E(SPL) family. • PCRs - regions of targeted PCR for confirmation of mRNA sequence.

In the model for *clockwork orange*, the protein domains on CWO sequences that are shared between vertebrates and insects include bHLH, basic-helix-loop-helix, Hairy Orange and an NLS. Additional protein domains found on the vertebrate DEC2 sequence including an Ala/Gly -rich domain and an HDAC interacting domain are not found on the insect orthologs. EST reads and directed PCR amplifications of the putative C-terminal region revealed stop codons in different positions, suggesting that siCWO may have several alternative splice variants. Our comparative analysis of the 3′ end of the gene across insects revealed an additional conserved domain not yet described for this gene, called *clockwork orange* C-tail Domain, CWOCD (Figure 4). The domain contains a conserved VPIFALH C-tail region that is present in all the putative orthologous insect sequences that were analyzed in this study. The 57–62 amino acids in this domain are largely conserved among insects (eg. ants and bees are similar at 52/58 sites in this region) and have little similarity to the Ala/Gly -rich domain in C-tail of vertebrate DEC2, the verterbrate orthdolog of CWO [33], [34]. Analysis of the CWOCD C-tail by Blastp algorithm (NCBI) showed that similar sequences are also present in Hairy and class b basic helix-loop-helix proteins (data not shown).

**Figure 5:**
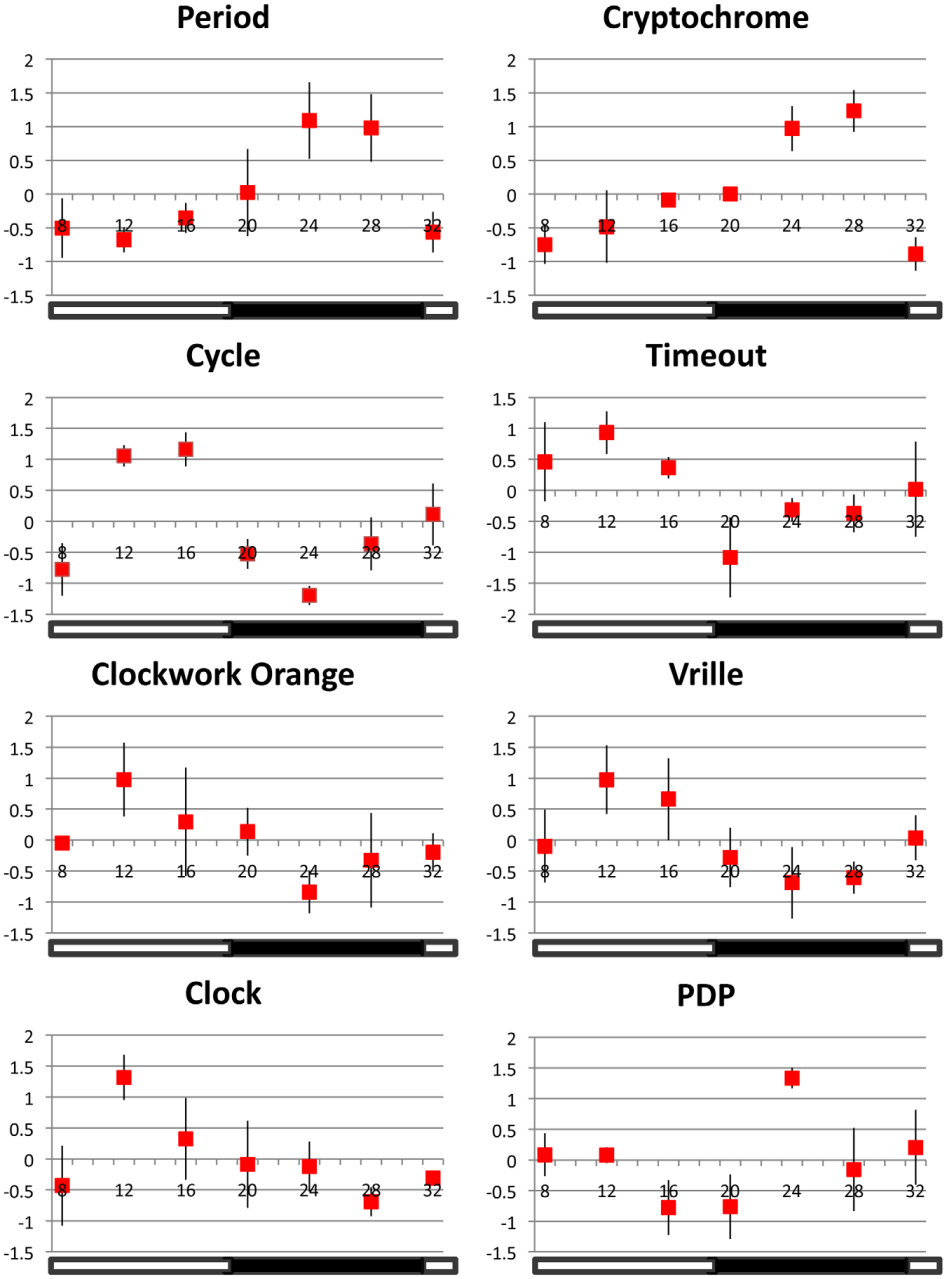
Relative gene expression patterns of 8 principle clock genes across colonies. Expression values for each data point (±SE) are plotted as the average relative expression level (ΔΔCt) across colonies (n = 3 colonies). Relative expression is calculated as the number of standard deviations above and below the mean value for all data points (across time). Standard error bars are calculated from variation across three colonies. The open stripe in the horizontal bar at base of the plot represents the daylight phase (12 hrs) and the solid stripe represents the dark phase (12 hrs) during the night. See Table 1 for additional details on clock gene expression.

**Figure 6:**
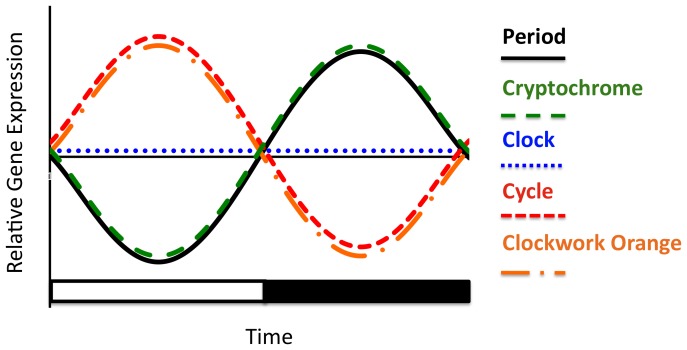
Summary representation of oscillation in mRNA for five core clock genes in fire ant brains under LD illumination. Shown are four genes (*SiPer, SiCry, SiCwo,* and *SiCyc)* that show significant oscillations and have significant correlations to the cosine model with R^2^adj≥0.5, and one gene, *SiClk*, which does not oscillate. Lines represent schematic cartoons of the actual oscillations. *SiPer* and *SiCry* cycle in the same phase and peak during the night and *SiCwo* and *SiCyc* cycle antiphase to *SiPer* and peak during the day. Not shown on the figure are the two transcription factors, *SiVri* and *SiPdp1*; *SiTim* was excluded because the expression of this gene was not consistent across nests.

The expression patterns of the eight principle clock genes suggest that the circadian clock of *S. invicta* functions similar to the honey bee clock (Figures 5, 6; Table 1). *SiPer* and *SiCry-m* oscillate with a similar phase–mRNA levels increase in the evening and peak during the dark phase (R^2^adj = 0.79, p = 0.003 and R^2^adj = 0.49, p = 0.015; respectively). *SiCyc* and *SiCwo* oscillate anti-phase to *SiPer* (R^2^adj = 0.54, p = 0.02 and R^2^adj = 0.60, p = 0.012 respectively). Weak oscillations for *SiVri* are similar in phase to *SiCyc* and *SiCwo* (R^2^adj = 0.63, p<0.0001). *SiTim*2 has significant, weak oscillations (R^2^adj = 0.55, p = 0.019) but patterns are not consistent within individual colonies. *SiPdp1* and *SiClk* do not show significant, consistent oscillations across colonies (R^2^adj = −0.2, p = 0.32 and R^2^adj = 0.19, p = 0.01 respectively). There are no significant differences over time for the control gene, EF1α (ANOVA F = 1.40, p = 0.29).

**Table 1:** Summary of gene expression data.

Gene	Peak Time[ZT]	ANOVA	Amplitude§	R^2/^R^2^adj [cosine model]	p-value† R^2^
**CLOCK**	6*	F = 1.55, p = 0.25	2.8	0.46/0.19	**0.010**
**CRY**	18	F = 6.33, **p = 0.01**	3.0	0.66/0.49	**0.015**
**CWO**	6	F = 7.27, **p = 0.02**	2.0	0.73/0.60	**0.012**
**CYCLE**	6	F = 2.45, **p = 0.00**	3.2	0.70/0.54	**0.020**
**PDP**	–	F = 2.06, p = 0.13	–	0.19/−0.2	0.320
**PERIOD**	18	F = 8.82, **p = 0.01**	2.4	0.86/0.79	**0.003**
**TIM**	6*	F = 1.34, p = 0.31	2.2	0.70/0.55	**0.019**
**VRILLE**	6	F = 1.26, p = 0.34	2.0	0.76/0.63	**0.000**

* Values not consistent across nests.

† p-values in **bold** are significant at α = 0.05.

§ Amplitudes calculated from peak/trough values of cosine model.

## Discussion

### The Molecular Clockwork in the Ant is Similar to that in the Honey Bee

The molecular dissection of the fire ant circadian clock reveals that ants have a mammalian-like clock with high similarity to the honeybee clock. Our study represents the most complete molecular characterization of a hymenopteran clock to date. Sequences of the eight principle clock genes are more closely related to the honeybee orthologs than to those of *Nasonia* and expression patterns of these genes suggest the molecular clockwork of ants utilizes similar mechanisms as the bee clock. In addition, the gene models from the fire ant show that all genes contain the necessary domains for the predicted function of the genes according to a mammalian-like clockwork and lack key components that characterize *Drosophila*-like clocks.

The fact that fire ant clock orthologs are more similar to honeybee clock orthologs than *Nasonia* in phylogenetic analyses is consistent with the monophyly of the Aculeata; i.e., the hypothesis that ants are more closely related to bees than to chalcidoid wasps [35]. Genome-wide BLASTp searches of fire ant proteins against protein databases indicate that 47% of *Solenopsis invicta* genes have the strongest similarity to apoid sequences and an additional 22% similar to *Nasonia* [30]. It is well documented that the bee clock is intricately connected to the social biology of this species [14], [18], [19], [36], [37]. Because ants and bees evolved sociality independently, it was not clear whether both clades evolved similar molecular mechanisms for regulating the circadian clock. Our results suggest that the clocks of ants and bees are regulated by the same mechanisms.

One diagnostic feature of the similarity in function of ant and bee clocks is found in the *cryptochrome* gene model. Cryptochromes belong to the photolyase gene family. Photolyases have a high affinity for complementary DNA strands and break certain types of pyrimidine dimers that arise when a pair of thymine or cytosine bases on the same strand of DNA become covalently linked. Proteins containing this domain also function as blue light photoreceptors that mediate blue light-induced gene expression and modulation of circadian rhythms. In *Drosophila*, the *cryptochrome* ortholog (*Cry-d*) functions as a photoreceptor in brain cells [1]. The light-dependent function of the gene requires a highly variable C-terminal domain that permits the interaction of the gene with TIM1 [*timeless*, a gene not found in the ant or bee genome] [38]. In mammalian-type cryptochromes (*Cry-m*), the C-terminal region is highly divergent from *Cry-d* orthologs. In contrast, *Cry-m* orthologs share three RD domains that are not found in *Cry-d* genes. These domains are responsible for nuclear localization and for the repression of CLK/BMAL transcription [2]. Our results show ant genomes encode the *Cry-m-*type cryptochrome with high sequence similarity and comparable expression profiles to the honeybee ortholog. Two lines of evidence suggest that the *Cry* gene is a functional component of the circadian mechanism in ants, like it is in bees and mammals. First, *Cry* mRNAs continue to oscillate in complete darkness [DD] in another ant species, *P. occidentalis* (unpublished data). Second, sequence similarity between ant and bee models of this gene is high [98%] which suggests that the function of this gene is conserved [1].

Another diagnostic feature is whether the CYC protein contains a transactivation domain. CYC (typically called BMAL1 in mammals) proteins are transcriptional factors with PAS-bHLH domains. In honeybees, amCYC is phylogenetically related to *Drosophila* CYC, but contains a highly conserved C-terminal transactivation domain that is found in mammalian BMAL proteins. In addition, honeybees lack the C-terminal region on amCLK that is responsible for transcriptional activity in dmCLK. Our results show that ant orthologs to CYC and CLK parallel the structural features of honeybee clocks. Thus, we would predict similarity in the temporal pattern of gene expression in ants and bees. In bees, brain transcript levels of *amCyc* oscillate nearly anti-phase to *amPer* while *amClk* does not oscillate [2], [27], [39]. In ants, two out of three nests showed strong oscillations in *siCyc* that were anti-phase to *siPer* oscillations. However, we also found a weak, non-significant trend in *siClk* under LD conditions. More data on the gene expression of *clock* in ants under DD conditions is necessary to determine whether the expression of this gene is under circadian influence in ants.

Our data provide the first report of qPCR gene expression patterns for *Vrille* and *Pdp*1, two conserved basic zipper transcription factors, in Hymenopterans. *SiVri* shows a possible daily oscillation antiphase to *SiPer* oscillations. *SiPdp1* does not appear to oscillate in the fire ant. The expression pattern of *SiVri* is similar to that found for aphids [40] and is not consistent with that seen in *Drosophila*, where transcripts for the gene are controlled by CLK/CYC complex. A whole brain microarray analyses suggest that *amVri* mRNA levels do not vary during the day in both nurse and forager honey bees sampled in DD, but this finding has not been yet validated with qPCR [27]. Additional studies are needed for determining if the pattern (and function) of *Vri* expression differ in bees and ants.

Overall, there is a potential difference in the expression of *Vri* but the remarkable similarity of ant and bee clocks supports the hypothesis that the Hymenoptera clock diverged from the basal insect clocks that contained both mammalian and *Drosophila* types of *Cry* and *Tim* [2]. Although the clocks of social hemimetabolous insects, i.e. termites, have not yet been studied in detail, the holometabolous social insects appear to utilize a molecular clockwork that generally functions similar to mammalian clocks and have lost the set of clock genes that were retained in *Drosophila* [2]. Our results will facilitate comparative sociogenomic analyses of circadian rhythms in the Aculeates. Understanding the similarities and differences in the regulation of the ant and bee clocks may give new insight into the role of circadian rhythms in regulating colony behavior and will help determine whether mechanisms of chronobiological plasticity are shared across social insects [11], [14], [17].

### A Novel Clockwork Orange Domain in Insects

Recent studies indicate the *clockwork orange* is an integral part of the circadian clockwork in Drosophila [31]–[33]. *Cwo* encodes a transcriptional repressor that is thought to compete with the CLK/CYC complex to bind to the E-box proteins [31]. The CWO protein also regulates itself by forming its own negative feedback loop and repressing the transcription of the *Cwo* gene. In *Drosophila*, CWO appears to function in regulating the amplitude of circadian oscillations in other core clock genes. The mammalian ortholog to *Cwo*, *Dec2* is involved in sleep length shifts [41]. As yet, we do not understand the role of CWO in hymenopteran insects.

To our knowledge, our results present the first gene-tree and model comparing *clockwork orange* across insects. Our analysis highlighted the presence of a novel domain in this gene that is present in insect CWO proteins but not the mammalian orthologs DEC1 and DEC2. The discovery of this conserved insect-specific domain in CWO contrasts with the overall similarity of social insect and mammalian orthologs of PER, CRY-m, CLK and CYC. This suggests that hymenopteran and *Drosophila* clocks may operate differently for some of the core feedback loops in the molecular clockwork but may share a mechanism for regulating the feedback loop of *clockwork orange*, the particular function of which is yet unknown for insects. Interestingly, the expression pattern of *SiCwo* in this study parallels patterns seen in the mouse, with high levels of *SiCwo* transcript during the day (peak at CT6) and lower levels during the night. This is similar to the oscillation seen in honey bees (peak at CT7; [27]) and in contrast to the pattern seen in *Drosophila* where *Cwo* transcripts peak later at ∼CT12 [31]–[33]. Thus, the structure of the *clockwork orange* gene in ants matches the structure found in insect *Cwo* genes and the expression pattern suggests a possible involvement in the positive loop of the clock.

*Clockwork orange* encodes a transcriptional repressor that inhibits CLK-mediated activation via interactions with PER [33]. In *Drosophila*, evidence suggests that CWO acts primarily in the late night to terminate CLK/CYC-mediated transcription of target genes. CWO creates its own negative feedback system, as it is one of the target genes regulated by the CLK/CYC complex through canonical E-box sequences [31]. The mammalian homologues (DEC1 and DEC2) appear to operate with a similar feedback mechanism. The feedback loop involving CWO is essential for the development of circadian rhythms in flies because CWO-deficient strains of flies show disruptions in oscillations of core clock genes and arrhythmic behavior [33]. In addition, the direct suppression of core clock genes through the CWO negative feedback loop helps generate and sustain high-amplitude oscillations [33].

The discovery that *clockwork orange* activity regulates the amplitude of the other core genes in the *Drosophila* clock underscores the complexity of the integrated positive and negative feedback loops that comprise the insect clock. Given the extensive studies of the E-box mediated negative feedback system [5], one of the key components of the circadian clock, the finding that the *clockwork orange* gene can regulate circadian expression via the E-box demonstrates that the molecular mechanism for circadian rhythms likely has redundancy among its components. The high conservation of the CWOCD sequence in the C-tail of the insects' CWO proteins suggest that this domain has a conserved but yet unknown function. The difference between the C-tail of insects and vertebrates provides yet another clue as to how evolution has shaped the diversity of circadian clocks and their regulation.

## Materials and Methods

### Development of Gene Models and EST Analyses

We developed gene models for five clock proteins in the fire ant *Solenopsis invicta*: *Cry, Cyc, Per, Clk and Cwo*. Selected genes contained domains known to differ across species and/or had adequate information known from multiple species. To determine gene models, we first ran TBLASTN using *Drosophila Cry, Cyc, Per, Clk or Cwo* proteins. Subsequently, ruby/bioruby scripts [http://bioinformatics.oxfordjournals.org/content/26/20/2617.short http://www.biomedcentral.com/1471-2105/10/221/abstract] were used to extract relevant subsets of the fire ant genome. Automated gene models were generated using MAKER2 [http://www.biomedcentral.com/1471-2105/12/491] and subsequently manually refined using Apollo [http://genomebiology.com/2002/3/12/research/0082; http://genome.cshlp.org/content/18/1/188.full]. Libraries of orthologous sequences were created by searching protein databases with the Blastp algorithm [http://nar.oxfordjournals.org/content/36/suppl_2/W5.short] and using siCRY, siCYC, siPER or siCLK protein sequences as a query. From the Blastp results, putative orthologs of CRY, CYC, PER or CLK were selected from species that are relatively well characterized (e.g. *Drosophila melanogaster* or the mouse) or of special interest (e.g. *Nasonia vitripennis*).

Swiss-Prot records for the mouse clock protein orthologs that have good annotation (marked regions) of the major protein domains were used to identify the protein domains on CRY, CYC, PER and CLK insect orthologs (mmCRY1 accession number: P97784, mmBmal1 accession number: Q9WTL8, mmPER1 accession number: O35973 and mmCLK accession number: O08785). Putative domains on the insect orthologous were identified using the EBI multiple sequence alignment CLUSTALX/CLUSTALW algorithm [42]. For each focal clock protein (CRY, CYC, PER or CLK) the protein models were aligned with the various species and the sequences corresponding to the protein domains on the mice proteins were marked on the insect orthologs. The sequences corresponding to each protein domain on the various orthologs were confirmed with the NCBI [43], SMART [44], [45], Pfam [46], ProSite [47] and InterPro [48] databases. Additional domains and motifs were delineated based on sequences reported in the literature, and for which the appropriate citation is provided. The EBI Global Alignment program [49] was used to determine the degree of amino acid residue identity/similarity between domains on the *S. invicta* protein models and corresponding amino acid sequences on orthologs from other animals. Domains were defined as conserved only if the amino acid sequence identity was ≥40% compared with the domain sequence on the mouse orthologs proteins [50].

Available EST sequences provided confirmation for most regions of the protein models. The “Translate tool” from the ExPASy proteomics server was used to predict the amino acid sequence encoded by each EST nucleotide sequence. Sequences that encode predicted polypeptides without stop codons in the middle were selected. The CLUSTALX/CLUSTALW algorithm was used to align the predicted EST amino acid sequences to the corresponding MAKER/Apollo protein model, and the protein models were corrected according to the amino acids predicted based on the EST sequences.

For CWO, a library of CWO ortholog sequences was compiled using the annotated CWOs found in [51]. The original library was selected using sequences >20% identity to the corresponding siCWO sequence. The protein domain sequences and conserved regions were identified as above. The Stockholm Bioinformatics Center's NucPred [http://www.sbc.su.se/~maccallr/nucpred/cgi-bin/single.cgi] was used to search for putative nuclear localization signals [NLS]. The NLS sequence was predicted in all CWO proteins present in the model. It is located inside bHLH domain with 100% identity in ortholog CWO sequences analyzed in this study. Additional domains and motifs were delineated based on relevant literature in which their biochemical function was defined [32], [34], [41].

Five EST sequences clustered together with the siCWO model. One of these clusters contained gaps inside the alignment caused by regions in an EST sequence that were not present in the Apollo model for siCWO. Translation of directed PCR in this region confirmed the Apollo model, suggesting that the EST with the extra regions in its sequence may represent an unprocessed mRNA of siCWO.

### Phylogenetic Analysis for the Clock Gene Proteins

Amino acid sequences from the open reading frames of the eight principle clock genes were aligned to orthologs found in Genbank and the ant genome database (now FOURMIDABLE [52] and references in [30], [53]–[57] ) using CLUSTALW in MEGA 5.0 (Table S1). Sequences of genes with highly variable C-tail regions were trimmed. Parsimony trees were constructed from the alignments using TNT phylogenetic software after exporting the data from SequenceMatrix. All analyses used 100 random addition runs with bootstrap values based on 250 replicates. For comparison, phylogenies were also constructed using maximum likelihood methods based on the JTT matrix-based model in MEGA 5.0. The robustness of the unrooted tree was assessed using bootstraps (1000 replicates).

### Gene Expression Analyses

Three *Solenopsis invicta* colonies were sampled from laboratory colonies housed at the USDA-ARS Center for Medical, Agricultural, and Veterinary Entomology in Gainesville, FL. Colonies were allowed to acclimate for 2 days in LD conditions (12 hours L/12 hours D) and on the third day, 20 foragers were collected from each colony at 7 timepoints (every 4 hours). Individuals were placed on dry ice immediately and remained frozen at −70°C until extraction. On the day of collection, samples were shipped overnight on dry ice. Total RNA was extracted from whole heads and pooled across 12 individuals for each source colony (Qiagen RNAez Micro Kit). Because ants were sampled in LD conditions, it is not possible to distinguish whether changes in gene expression over the day are a result of endogenous circadian rhythms, are influenced by gene expression responses to exogenous light patterns, or both.

Fire ant-specific primers were designed from exon-coding regions to amplify 60–120 bp regions for qPCR analyses. cDNA was synthesized from extracted total RNA preps using ABI TaqMan Gold Reverse Transcriptase reagents and random hexamers. The 10 uL reactions included 1.0 uL of RNA with 1 X TaqMan RT Buffer, 5.5 mM 25 mM MgCl2, 500 uM of each of the deoxyNTPs, 2.5 uM of the Random Hexamer primers, 0.4 U/uL of RNase Inhibitor and 1.5 U/uL of MultiScribe Reverse Transcriptase. Reactions were performed in triplicate for each sample at each time point. All reactions were run at 25°C for 10 minutes, 48°C for 30 minutes, 95°C for 5 minutes, and then stored at −20°C until quantitative PCR. For each cDNA replicate, gene expression was assayed on an ABI 7900 HT instrument using ABI Taqman Gold reagents with gene specific primers (Table S2). The 25 uL qPCR reactions for each gene included 3.5 uL of template cDNA with 1 X TaqMan Buffer A, 5.5 mM 25 mM MgCl2, 200 uM of 10 mM deoxyATP, 200 uM of 10 mM deoxyCTP, 200 uM of 10 mM deoxyGTP, 400 uM of 20 mM deoxyUTP, 100 nM of probe, 200 nM of each primer, 0.01 U/uL of AmpErase UNG and 0.025 U/uL of AmpliTaq Gold DNA Polymerase. To standardize clock gene expression, EF1α was used as a control for each cDNA replicate. The 25 uL qPCR reactions for the control included 1.5 uL of template cDNA and the same reaction mixture described above. The following primers were used for the control gene: SiEF1α Forward: GGCTCTGAGGGAGGCTTT, SiEF1α Reverse: CGGAGATGTTCTTCACGTTGAA, SiEF1α Probe: CTCGCGATAACGTCG. Real-time PCR reactions for target genes and SiEF1α were performed under the following conditions: 2 min at 50°C for one cycle, 10 min at 95°C for one cycle, 15 sec at 95°C, 1 min at 60°C, for 45 cycles. Data were analyzed using SDS 2.1 software and quantification of relative mRNA levels and standard errors were calculated using the ΔΔCt method (ABI User Bulletin).

Gene expression patterns for each colony were analyzed separately. Significant differences in gene expression over time were tested using a two-way ANOVA [colony, timepoint] for each gene, including EF1α in STATA. Changes in gene expression over time were compared to a cosinor model to determine how closely the oscillations fit a generalized circadian model using MATLAB and the equation detailed in [12]. Ten million simulations were run starting with random seeds. The top 400 values that best fit the cosinor model were averaged to obtain the R^2^ and amplitude values. Significance values for the fit to the cosine model were calculated in MATLAB. R^2^ adjusted was calculated from the estimates of R2 according to [12]. Oscillations in genes were considered significant if both ANOVA p-values and R^2^ were ≤0.05 and R^2^ values explained a large portion of the variance (R^2^adj≥0.5).

## Supporting Information

Figure S1 **Additional phylogenetic trees.**
(PPTX)

Figure S2 **Additional gene models.**
(PPTX)

Table S1 Sequences used for phylogenetic alignments and gene models.
(DOCX)

Table S2 Primers used in expression analyses.
(DOCX)

Table S3 Photo credits for gene models.
(DOCX)
