# Supplementary material for: The Molecular Clockwork of the Fire Ant Solenopsis invicta
Source: PLoS One. 2012 Nov 13;7(11):e45715. doi: 10.1371/journal.pone.0045715 (PMC3496728; doi:10.1371/journal.pone.0045715)
Supplement: Table S1 — Sequences used for phylogenetic alignments and gene models. (DOCX) [file pone.0045715.s003.docx]

Clockwork orange

| Protein name | Abbreviation | Organism | Database | Accession number | Length (aa) |
| --- | --- | --- | --- | --- | --- |
| Clockwork orange | siCWO | *Solenopsis invicta*  (Fire ant) | NCBI | JX948387 | 404 |
| Clockwork orange | amCWO | *Apis mellifera*  (Western honey bee) | NCBI | XP_001121958.1 | 400 |
| Clockwork orange | tcCWO | *Tribolium castaneum*  (Rust Red Flour Beetle) | NCBI | XP_001812240.1 | 409 |
| Clockwork orange | agCWO | *Anopheles gambiae*  (African malaria mosquito) | --- | ---- | 588 |
| Clockwork orange | dmCWO | [*Drosophila melanogaster* (Fruit fly)](http://www.uniprot.org/taxonomy/7227) | Swiss-Prot | Q9VGZ5 | 698 |
| Clockwork orange | dvCWO | *Drosophila virilis*  (Fruit fly) | NCBI | XP_002053032.1 | 666 |
| Clockwork orange | deCWO | *Drosophila erecta*  (Fruit fly) | NCBI | XP_001980677.1 | 700 |
| Clockwork orange | dppCWO | *Drosophila pseudoobscura pseudoobscura*  (Fruit fly) | NCBI | XP_001358195.2 | 704 |
| Clockwork orange1 | dgCWO1 | *Drosophila grimshawi*  (Fruit fly) | NCBI | XP_001990050.1 | 667 |
| Clockwork orange | dmoCWO | *Drosophila mojavensis*  (Fruit fly) | NCBI | XP_001998538.1 | 675 |
| Clockwork orange | daCWO | *Drosophila ananassae*  (Fruit fly) | NCBI | XP_001952949.1 | 690 |
| Clockwork orange | lhCWO | *Linepithema humile*  (Argentine ant) | -- | -- | 403 |
| Clockwork orange | pbCWO | *Pogonomyrmex barbatus*  (Red harvester ant) | -- | -- | 380 |
| Clockwork orange | hsCWO | *Harpegnathos saltator*  (Jumping ant) | -- | -- | 386 |
| Clockwork orange | cfCWO | *Camponotus floridanus*  (Carpenter ant) | -- | -- | 380 |
| Clockwork orange | aeCWO | *Acromyrmex echinatior*  (Leaf-cutting ant) | -- | -- | 383 |
| Clockwork orange | acCWO | *Atta cephalotes*  (Leafcutter ant) | -- | -- | 382 |
| BHLHB3 | mmDEC2 | *Mus musculus*  (Domestic mouse) | NCBI | BAB21503.1 | 410 |
| BHLHB3 | drDEC2 | *Danio rerio* (Zebrafish) | NCBI | BAE72667.1 | 421 |

Clock

| Protein name | Abbreviation | Organism | Database | Accession number | Length (aa) |
| --- | --- | --- | --- | --- | --- |
| Clock | siCLK | *Solenopsis invicta*  (Fire ant) | NCBI | JX948388 | 673 |
| Clock | amCLK | *Apis mellifera*  (Western honey bee) | NCBI | XP_394233.4 | 724 |
| Clock | nvCLK | *Nasonia vitripennis*  (Jewel wasp) | NCBI | XP_001599257.2 | 728 |
| Clock | apCLK | *Anthereae pernyi*  (Giant silk moth) | Swiss-Prot | Q6VRU6 | 611 |
| Clock | mmCLK | *Mus musculus*  (Domestic mouse) | Swiss-Prot | O08785 | 855 |
| Clock | dmCLK | [*Drosophila melanogaster* (Fruit fly)](http://www.uniprot.org/taxonomy/7227) | Swiss-Prot | O61735 | 1027 |

Cryptochrome

| Protein name | Abbreviation | Organism | Database | Accession number | Length (aa) |
| --- | --- | --- | --- | --- | --- |
| Cryptochrome | siCRY | *Solenopsis invicta*  (Fire ant) | NCBI | JX948389 | 590 |
| Cryptochrome -m | amCRY-m | *Apis mellifera* (Western honey bee) | NCBI | NP_001077099.1 | 570 |
| Cryptochrome-1 | nvCRY-1 | *Nasonia vitripennis* (Jewel wasp) | NCBI | XP_001606405.2 | 627 |
| Cryptochrome-1 | mmCRY-1 | *Mus musculus*  (Domestic mouse) | Swiss-Prot | P97784 | 606 |
| Cryptochrome 1 | dpCRY1 | *Danaus plexippus* (Monarch butterfly) | Swiss-Prot | Q2TJN5 | 534 |
| Cryptochrome1-D | dmCRY1-d | *Drosophila melanogaster*  (Fruit fly) | Swiss-Prot | O77059 | 542 |

Cycle

| Protein name | Abbreviation | Organism | Database | Accession number | Length (aa) |
| --- | --- | --- | --- | --- | --- |
| Cycle | siCYC | *Solenopsis invicta*  (Fire ant) | NCBI | JX948390 | 850 |
| Cycle | amCYC | *Apis mellifera* (Western honey bee) | NCBI | XP_001121441.2 | 925 |
| Cycle | nvCYC | *Nasonia vitripennis* (Jewel wasp) | NCBI | XP_001602684.2 | 823 |
| BMAL | apBMAL | *Anthereae pernyi*  (Giant silk moth) | Swiss-Prot | Q6VRU5 | 589 |
| BMAL | mmBMAL | *Mus musculus*  (Domestic mouse) | Swiss-Prot | Q9WTL8 | 632 |
| Cycle | dmCYC | *Drosophila melanogaster*  (Fruit fly) | Swiss-Prot | O61734 | 413 |

Period

| Protein name | Abbreviation | Organism | Database | Accession number | Length (aa) |
| --- | --- | --- | --- | --- | --- |
| Period | nvPER | *Solenopsis invicta*  (Fire ant) | NCBI | JX948386 | 1089 |
| Period | amPER | *Apis mellifera*  (Western honey bee) | Swiss-Prot | Q9NDF3 | 1124 |
| Period | nvPER | *Nasonia vitripennis* (Jewel wasp) | NCBI | XP_001604906.2 | 1145 |
| Period | dpPER | *Danaus plexippus* (Monarch butterfly) | Swiss-Prot | Q7Z0C9 | 1056 |
| Period | mmPER1 | *Mus musculus*  (Domestic mouse) | Swiss-Prot | O35973 | 1291 |
| Period | dmPER | *Drosophila melanogaster*  (Fruit fly) | Swiss-Prot | P07663 | 1224 |
| Period | apPER | *Anthereae pernyi*  (Giant silk moth) | Swiss-Prot | Q17062 | 849 |
