# Supplementary material for: The Molecular Clockwork of the Fire Ant Solenopsis invicta
Source: PLoS One. 2012 Nov 13;7(11):e45715. doi: 10.1371/journal.pone.0045715 (PMC3496728; doi:10.1371/journal.pone.0045715)
Supplement: Table S2 — Primers used in expression analyses. (DOCX) [file pone.0045715.s004.docx]

| **Gene** | **Forward Primer** | **Reverse Primer** | **Probe** |
| --- | --- | --- | --- |
| **SiClock** | GGGTACGGGACGTTTGCA | CGCACTGTCGGTTATGGATAATT | ACTCCTCAGCTTATCC |
| **SiCry** | GGCCGCGTGCGAGAT | ACCACCGAGATACCGAGTTCCT | ACAATATCTCGGCCCTCT |
| **SiCWO** | GCGATCCGCCACATGAA | CGGATGAGTGCTTGGTATCTT | CATCTTCAAGGCCTCC |
| **SiCyc** | TGCGCGGAGATCCTTCTT | AACGGACCCCGTCGACTT | TGTCGGATGAAGCGC |
| **SiEFIa** | GGCTCTGAGGGAGGCTTT | CGGAGATGTTCTTCACGTTGAA | CTCGCGATAACGTCG |
| **SiPDP** | CGCTTGGTAACTGGTTCCAAA | AGCCAGCTTCATAAAATCAACAATAC | TAGACCCGCTGGTCCCTGATGCC |
| **SiPer** | GGAATGGCAGATACAGGTTTGAC | GCGGTCTGCGAAAGTTTACC | TGGCGTCCGCTCGATACCCG |
| **SiTim** | GAGCCGATAATGATGCTACTGTTC | CCACTATGCCTGACGCATGA | TGATGAGGTATTATTTGCG |
| **SiVrille** | AATTCGAGGCTGGTATTTCAAAGA | CGAGGGTGAAGGCGAGAAG | CGGACAAACCCGTGAGCACGC |
