# Supplementary material for: The Molecular Clockwork of the Fire Ant Solenopsis invicta
Source: PLoS One. 2012 Nov 13;7(11):e45715. doi: 10.1371/journal.pone.0045715 (PMC3496728; doi:10.1371/journal.pone.0045715)
Supplement: Table S3 — Photo credits for gene models. (DOCX) [file pone.0045715.s005.docx]

**Photo credits for gene models:**

*Solenopsis invicta -* [http://web.biosci.utexas.edu/fireant/index.html](http://www.google.com/url?q=http%3A%2F%2Fweb.biosci.utexas.edu%2Ffireant%2Findex.html&sa=D&sntz=1&usg=AFQjCNHgtFlHvtwMkxDJyIaHQJSx9C50zg)

*Apis mellifera -* [http://www.devbio.biology.gatech.edu/?page_id=2799](http://www.google.com/url?q=http%3A%2F%2Fwww.devbio.biology.gatech.edu%2F%3Fpage_id%3D2799&sa=D&sntz=1&usg=AFQjCNG4x-Z_Ew0S-ZxSTJJNMsD4tYi25g)

*Tribolium castaneum -* [http://myrmecos.net/insects/Tribolium5.html](http://www.google.com/url?q=http%3A%2F%2Fmyrmecos.net%2Finsects%2FTribolium5.html&sa=D&sntz=1&usg=AFQjCNGPRgv29SYB73BNvV7u-db0eKlaOA)

*Drosophila melanogaster* - [http://www.noldus.com/content/drosophila-activity-monitoring-system](http://www.google.com/url?q=http%3A%2F%2Fwww.noldus.com%2Fcontent%2Fdrosophila-activity-monitoring-system&sa=D&sntz=1&usg=AFQjCNHWxXXXB0sx5zYLddTgew_EZYeztA)

*Mus musculus* - [http://www.mundoplagas.com.ar/servicios-control-plagas-fumigaciones-buenos-aires/desratizaciones/](http://www.google.com/url?q=http%3A%2F%2Fwww.mundoplagas.com.ar%2Fservicios-control-plagas-fumigaciones-buenos-aires%2Fdesratizaciones%2F&sa=D&sntz=1&usg=AFQjCNGeAr5e3LRAlhvSR6wzdDQ7rGn1nQ)

*Danio rerio* - [http://ensembl.fugu-sg.org/Danio_rerio/index.html](http://www.google.com/url?q=http%3A%2F%2Fensembl.fugu-sg.org%2FDanio_rerio%2Findex.html&sa=D&sntz=1&usg=AFQjCNHE15QJzwGSYWF0HBwpPWETfICREQ)

*Anthereae pernyi -* [http://www.thefreedictionary.com/Pernyi+moth](http://www.google.com/url?q=http%3A%2F%2Fwww.thefreedictionary.com%2FPernyi%2Bmoth&sa=D&sntz=1&usg=AFQjCNFYxgCk1EAQZKiODQHquZfDrD1H9Q)

*Danaus plexippus* - [http://fohn.net/monarch-butterfly-pictures/](http://www.google.com/url?q=http%3A%2F%2Ffohn.net%2Fmonarch-butterfly-pictures%2F&sa=D&sntz=1&usg=AFQjCNHFb3tiBbt_1hGk29VOU0tCv9uaSg)

*Nasonia vitripennis -* (**not original source**) [http://mustangforums.com/forum/off-topic/329886-medium-sized-blue-bug.html](http://www.google.com/url?q=http%3A%2F%2Fmustangforums.com%2Fforum%2Foff-topic%2F329886-medium-sized-blue-bug.html&sa=D&sntz=1&usg=AFQjCNE1DhSnmwoufoHjxhweIxengBfUlw)
